# Open or closed: pH modulation and calcification by foraminifera

**DOI:** 10.1126/sciadv.adq8425

**Published:** 2025-05-02

**Authors:** Daniel François, Gert-Jan Reichart, Lennart J. de Nooijer

**Affiliations:** ^1^ Department of Ocean Systems, NIOZ Royal Netherlands Institute for Sea Research and Utrecht University, Texel, Netherlands.; ^2^ Department of Earth Sciences, Faculty of Geosciences, Utrecht University, Utrecht, Netherlands.

## Abstract

Marine calcifying organisms precipitate their shells either in equilibrium with seawater or under strict biological control. Here, we show that these two options represent two ends of a spectrum. In species with a more “closed” system, rates of H^+^ removal and Ca^2+^ uptake are high and exceed the amount of ions required for calcification. This explains the relatively low Mg/Ca of the calcite of this species by dilution of the [Mg^2+^] in the calcifying fluid. Conversely, in species with a more open system, the H^+^ and Ca^2+^ fluxes are lower, with more seawater exchanged between the environment and calcifying fluid, explaining the relatively high Mg/Ca in these foraminifera. In either of these species, mitochondria were found to be located at the site where the Ca^2+^/H^+^ exchange takes place and the mitochondrial density aligned with the rate of pumping. These findings highlight the crucial role of transmembrane transporters and mitochondria in foraminifera calcification and explain the species-specific elemental signatures.

## Teaser

Microenvironment pH control varies across foraminiferal species, explaining species-specific sensitivity to acidification.

## INTRODUCTION

Marine calcifying organisms play a crucial role in the balance of seawater carbonate chemistry by absorbing and storing carbon in the deep ocean through calcification and the biological pump mechanism. Globally, pelagic calcium carbonate (CaCO_3_) production by coccolithophores, foraminifers and pteropods ranges from 0.7 to 4.7 Gt C year^−1^ (*1*–*4*), with an export rate of approximately 0.6 ± 0.4 Gt C year^−1^ toward the seafloor (*3*). These calcifying taxa have distinct calcification mechanisms, and therefore, they respond differently to environmental changes, including ocean acidification (OA), which reduces the concentration of carbonate ions necessary for calcification. Among them, foraminifera can be relatively resilient to OA (*5*), particularly in their benthic forms. However, their responses vary between species, posing a challenge in predicting their net contribution to the future carbonate budget (*5*–*9*). This is of crucial importance for biogeochemical modeling since they are now estimated to account for 25% of CaCO_3_ global production [estimated at 1.4 Gt of CaCO_3_ year^−1^ (*10*) and up to half of the global CaCO_3_ sedimentation at the sea floor (*11*, *12*)].

Calcification has arisen independently multiple times over the course of foraminiferal evolution (*13*), and physiological responses to carbon perturbations likely reflect the degree of control each species has in regulating the composition of the calcifying fluid. For instance, the ability to regulate the pH of the calcifying fluid (pH_cf_) and magnesium/calcium (Mg^2+^/Ca^2+^) at the site of calcification (SOC) could aid in tolerating changes in seawater carbonate chemistry (*14*), but differences among species remain largely unknown (*15*–*18*). While removal of Mg^2+^ is unlikely as the sole mechanism to the decrease in fluid Mg/Ca ratios (*14*, *19*), the transport of H^+^ (out) and Ca^2+^ (in) is believed to be coupled to maintain electroneutrality (*18*), influencing the chemical composition of the calcifying fluid, hence the calcite Mg/Ca ratios.

Other factors that may influence calcification and element incorporation include Rayleigh fractionation (*20*), vacuolization, and/or seawater leakage to the SOC and involvement of mitochondria (*21*–*25*). The latter is relevant since mitochondria might serve as a (temporary) storage site for the Mg^2+^ and Ca^2+^ extracted from the SOC as well as providing the energy for H^+^/Ca^2+^ exchange (*19*, *22*, *23*, *25*).

Here, we test the relationship of H^+^ transport and biological pH_cf_ regulation using fluorescence microscopy by comparing the pH microenvironment of two species with contrasting calcite chemistries [*Ammonia tepida* and *Heterostegina depressa*, with Mg/Ca_calcite_ of 1 to 7 and 110 to 140 mmol/mol, respectively (*23*, *26*)]. In addition, the specificity of H^+^/Ca^2+^ was tested, and mitochondrial networks were labeled and compared to the activity of transporters. The results are the basis for a model that describes Mg^2+^ incorporation into calcite as a function of pH regulation and mitochondrial activity.

## RESULTS

### Microenvironment pH control varies between low- and high-Mg/Ca species

**Fig. 1.**
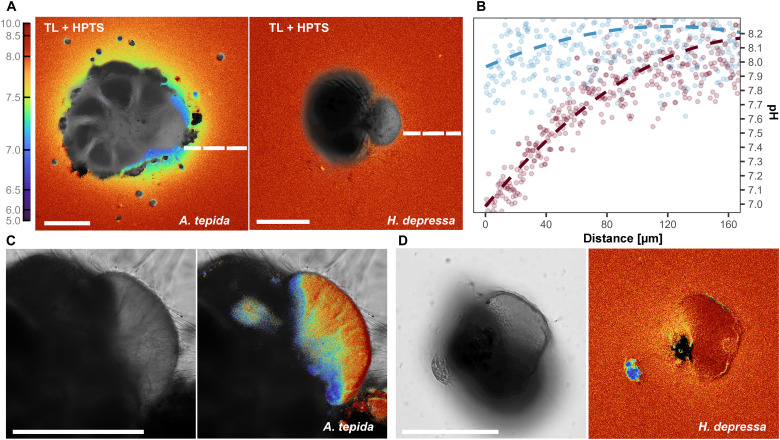
Activity of V-type ATPases among species during calcification. (**A**) pH maps calculated by dividing λ^405^em/λ^488^em + λ^405^em (λem = 510 to 560 nm) for each pixel and (**B**) the translated, spatially integrated change in pH measured over the white dashed lines of calcifying specimens of *A. tepida* (red line) and *H. depressa* (blue line) shown in (A). (**C**) pH maps of the site of calcification of *A. tepida* and (**D**) *H. depressa*.

Fluorescence labeling of seawater pH (Fig. 1A) revealed that pH decrease in the microenvironment surrounding calcifying *A. tepida* is 3.5 times lower compared to *H. depressa* (*t* test, *P* < 0.01, *t* = 11.9, df = 5.1), with seawater pH decreasing by 0.93 ± 0.12 and 0.26 ± 0.05 pH units, respectively (Table 1). Accordingly, the pH at the SOC is higher in *A. tepida* (~9.5) than in *H. depressa* (~8.5). These differences were inversely correlated to the duration of the time interval within which chamber formation was completed. Chamber formation takes the least amount of time for *A. tepida* (5.74 ± 0.57 hours) and last the longest for *H. depressa* (18.44 ± 3.89 hours) (*t* test, *P* < 0.01, *t* = −6.5, *df* = 3) (Table 1). For *A. tepida*, the strongest pH decrease was achieved approximately 80 min after the observed start of chamber formation and, for *H. depressa*, after about 340 min.

**Table 1.** Summary of pH imaging observations and calculated outward proton fluxes during chamber formation of *A. tepida* and *H. depressa*. The average seawater pH at 100- to 110-μm distance from the foraminiferal shell is 8.16 pH units (*n* = 10). The values in bold represent the mean values of the five individuals. ND, not determined. Id, identification. Ind, individual.

Species	Id.	Test size (μm)	Chamber size (μm)	pH microenvironment (seawater scale)	ΔpH	Local H^+^ flux (nmol/cm^2^/s)	Total H^+^ flux (pmol)	Time (hours)
*A. tepida*	Ind. 1	233	81	6.90	0.84	2.97	ND	ND
	Ind. 2	105	50	7.15	1.13	2.57	1940	5.33
	Ind. 3	94	40	7.35	0.85	2.96	1447	5.40
	Ind. 4	161	93.6	7.23	0.92	2.58	8364	6.56
	Ind. 5	184	90.4	7.15	0.91	3.70	9661	5.65
	**Average**	**155**	**71**	**7.16**	**0.93**	**2.96**	**5353**	**5.74**
*H. depressa*	Ind. 1	193	81	8.07	0.20	0.07	598	24.00
	Ind. 2	141	89	8.05	0.24	0.22	1470	15.00
	Ind. 3	134	89	7.95	0.31	0.18	1386	17.00
	Ind. 4	209	102	8.01	0.25	0.13	1340	17.75
	Ind. 5	251	125	7.83	0.30	0.11	ND	ND
	**Average**	**186**	**97**	**7.98**	**0.26**	**0.14**	**1198**	**18.44**

### H^+^/Ca^2+^ exchange system displays high specificity

**Fig. 2.**
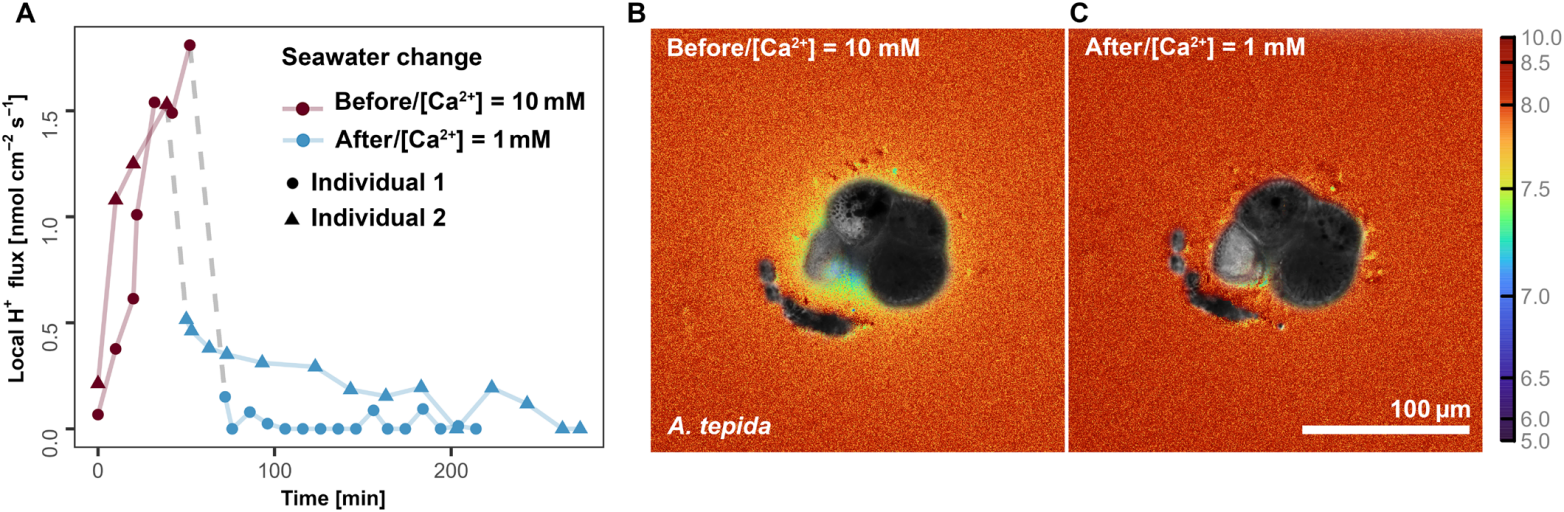
Specificity of H^+^/Ca^2+^ exchange system in *A. tepida*. (**A**) Calculated local H^+^ flux for *A. tepida* before (red line) and after (blue line) change of seawater [Ca^2+^]. The pH maps calculated by dividing λ^405^em/λ^488^em + λ^405^em (λem = 510 to 560 nm) for each pixel before (**B**) and after (**C**) the change of seawater [Ca^2+^]. Complete dataset is available in dataset S1.

To investigate the specificity of the H^+^/Ca^2+^ exchange, seawater calcium concentrations were reduced to 10% of the original in the middle of calcification events (Fig. 2, A to C). The seawater calcium concentration was altered well after the calcification site had formed and proton pumping activity was noted (Fig. 2A). The results show that reducing seawater [Ca^2+^] considerably affect the activity of H^+^ pumps (Fig. 2, A to C). Specifically, a significant 93% decrease in proton pumping activity was observed, from 1.67 to 0.12 nmol cm^−2^ s^−1^, compared to the highest value observed before the reduction in seawater [Ca^2+^] (data available in table S1).

### Mitochondrial distribution during calcification

**Fig. 3.**
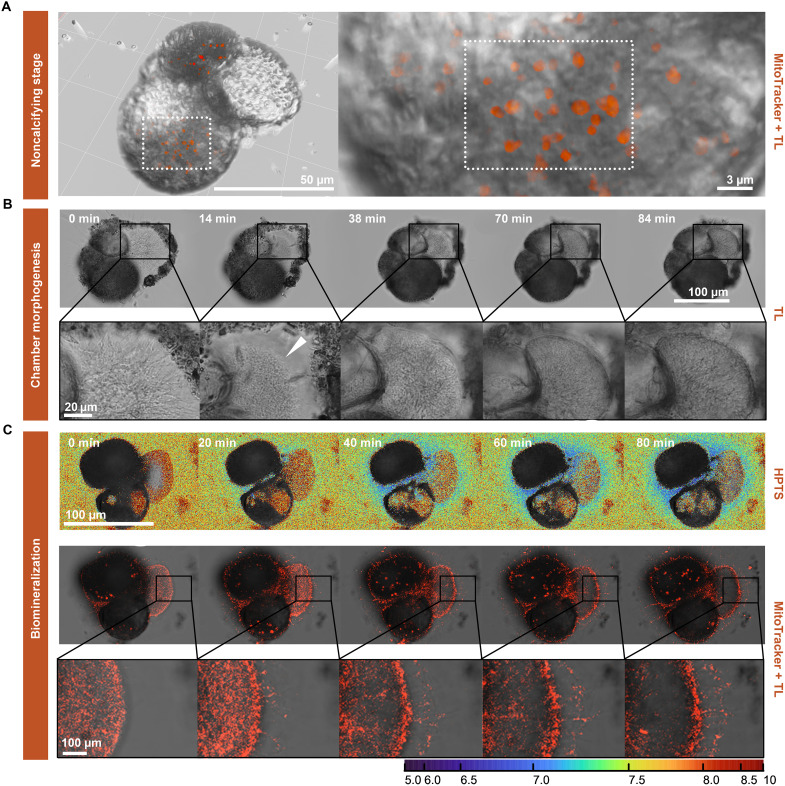
Colocalization of mitochondrial networks and pH maps in *A. tepida*. (**A**) Three-dimensional volume rendering of labeled active mitochondria using MitoTracker Red (200 nM) during the noncalcifying stage. (**B**) Successive stages of chamber morphogenesis (transmitted light) of calcifying specimens of *A. tepida* (individual 8). (**C**) Recorded time series of fluorescence labeling of active mitochondria with MitoTracker Red (200 nM) and seawater pH maps with HPTS (20 μM) of calcifying *A. tepida*. The false-color scale bar represents seawater pH changes calculated by dividing λ^405^em/λ^488^em + λ^405^em (λem = 510 to 560 nm) for each pixel. The artificial red color represents mitochondria labeling.

Preceding chamber formation, specimens of *A. tepida* (Table 1) retract and reduce the activity of their long, thin rhizopodia (Fig. 3A). Once settled at a location, a distinct cytoplasmic bulge expands, carrying the mitochondria outside the test, which concentrates at the edge of an organic scaffolding (Fig. 3B). After the shape of the calcifying chamber is outlined, the pseudopodia form a dense network in a fan-like arrangement that envelops the zone in which the calcifying chamber wall will form (Fig. 3C). Approximately 20 min after the start of this phase, the pH outside this network gradually decreases by 0.93 ± 0.12 units (Fig. 3C). During this phase, time-lapse microscopy of labeled mitochondria demonstrates that they are moving outward throughout radial extensions of the pseudopodia into the formed biomineralization framework (*27*). Hence, the observed decrease in ambient pH is paralleled by an increase in the density of mitochondria near the SOC (Fig. 3C). After approximately 80 min, the ambient pH was lowest, and mitochondrial density was also highest (Fig. 3C). At the end of chamber formation (after approximately 5.7 hours), pseudopodia and mitochondria are retracted again, and pH in the foraminifer’s microenvironment is similar to ambient values. After calcification of the chamber is completed, specimens start moving again, and the original long and sparse pseudopodia, characteristic of a dynamic stage, appear with some visible mitochondria (Fig. 3A).

**Fig. 4.**
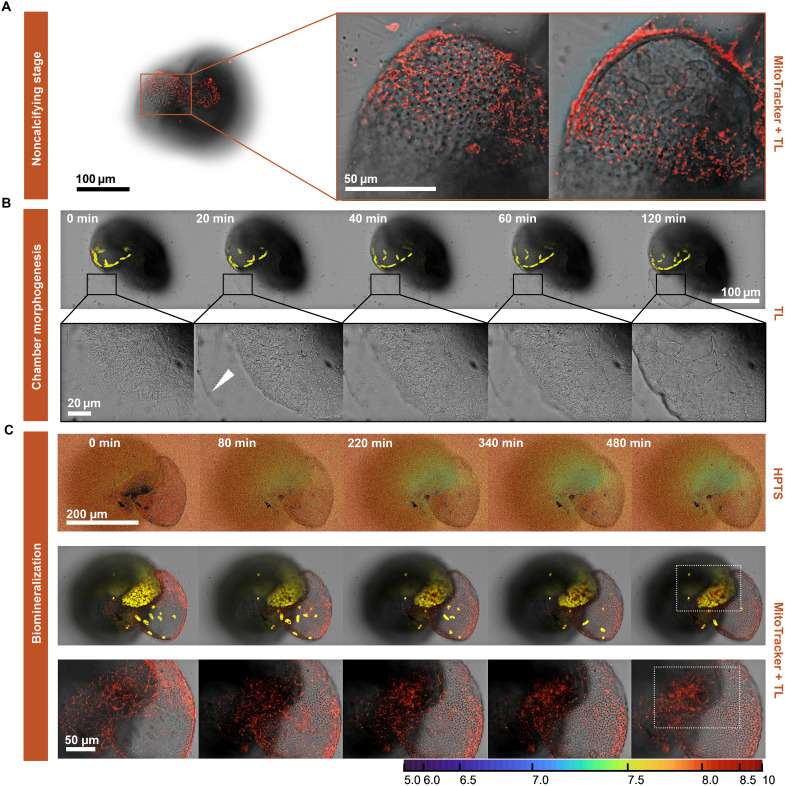
Colocalization of mitochondrial networks and pH maps in *H. depressa*. (**A**) Fluorescence labeling of active mitochondria in *H. depressa* using MitoTracker Red (200 nM) during the dynamic stage. (**B**) Successive stages of chamber morphogenesis (transmitted light) of calcifying specimens of *H. depressa*. (**C**) Recorded time series of fluorescence labeling of active mitochondria with MitoTracker Red (200 nM) and seawater pH maps with HPTS (20 μM) of calcifying *H. depressa* (individual 13). The false-color scale bar represents seawater pH changes calculated by dividing λ^405^em/λ^488^em + λ^405^em (λem = 510 to 560 nm) for each pixel. The artificial red color represents mitochondria labeling. The artificial yellow color represents autofluorescence from symbionts (λem = 661 to 672 nm).

At the location where specimens of *H. depressa* are, individuals rotate inside an ectoplasm sheath to open space for a new chamber, marking the beginning of calcification. This is followed by an expansion of the cell’s protoplasm (the “bulge”; Fig. 4B), outlining the chamber shape (1.5 hours; Fig. 4B). As observed in *A. tepida*, mitochondria are carried outside the test, accumulating near the cell membrane. In a second stage, the individual forms the organic templates recognized by the appearance of pore plates (after approximately 30 min; Fig. 4B). During this process, pseudopodia and mitochondrial activity decrease inside the test, and the symbionts start to colonize the forming chamber (Fig. 4C). In the following minutes, ambient pH gradually decreases to a minimum of 7.98 ± 0.10 (with pH, on average, 0.26 ± 0.05 lower compared to that of the seawater; Table 1), and calcite deposition starts (Fig. 4C). At this later stage, MitoTracker labeling shows that mitochondria are characteristically found adjacent to the cell wall in association with the outer protoplasmic layer (Fig. 4C).

## DISCUSSION

### Proton pumping rates differ between foraminiferal species

The sequence of events leading to a dedicated space/SOC (Figs. 3B and 4B) was similar for the species studied here and that described before for other species (*27*–*30*). In summary, a cytoplasmic bulge expands, forming a specialized membrane-bound compartment in which calcite crystal nucleation takes place. Once this network is formed, the pH outside the calcifying chamber decreases, and the pH inside the SOC increases for both species, albeit to a different degree (Fig. 1, A to F).

In *A. tepida*, the large internal-external pH difference (>9 versus <7) is in accord with previous measurements (*16*, *18*) and suggests that calcification proceeds in a tightly controlled calcifying reservoir (*17*, *18*). The chemistry of the calcifying fluid has an inorganic carbon chemistry markedly different from that of the surrounding seawater, explaining the observed decoupling between lowered ambient saturation and continued calcification for this foraminiferal genus (*31*–*34*). This is in line with the dominance of *A. tepida* in tidal flats and estuaries where carbonate chemistry (and conditions in general) fluctuate strongly on daily and seasonal scales, e.g., 6.8 to 9.1 pH units (*35*).

In contrast, the pH increase at the SOC (~0.45 pH units) and decrease outside (~0.26 pH units), observed in the microenvironment of calcifying *H. depressa* and measured here, were less pronounced. This suggests a more open SOC with less active pumping, which explains the high sensitivity of this species to acidic conditions in both in situ (*6*, *36*) and laboratory-controlled conditions (*37*). Although the observed decrease in external pH seems to reflect respiration rather than active proton pumping, we measured here a small but distinctly different pH during calcification in *H. depressa* (0.26 ± 0.05 pH units) compared to what is observed only during respiration (~0.1 pH units; fig. S1, A to C). For other species, the respiration-related offset in pH is quite similar ~0.1 pH units (*38*). We note that during the time series, specimens were kept in the dark for the complete duration of the experiment (~18 hours), with lasers active for only 20 s every 30 min during acquisition. Therefore, the influence of symbionts and photosynthesis on pH measurements are considered negligible here.

The composition of the shell of *A. tepida* is similar to that of planktonic foraminifera, not only in terms of Mg/Ca but also in other elements (*13*). This resemblance is also evident from the comparable microstructures, such as typical bilamellar layering with alternating high- and low-El banding (*39*–*42*). This suggests that the mechanisms by which all these foraminifera produce their calcite are very similar, implying that the responses observed in *A. tepida* are likely applicable to other low-Mg/Ca species too. We note, however, that the existence of a microenvironmental pH control system is yet to be confirmed for planktonic species. If this is the case, then the strong difference in internal-external pH observed during foraminiferal calcification in *A. tepida* (Table 1 and Fig. 1) supports the notion that low-Mg/Ca foraminifera may maintain or even enhance calcification in response to elevated *p*CO_2_ levels (*18*). This is because, although increased pumping is required to establish an adverse pH gradient under high *p*CO_2_ conditions, it is counteracted by the higher availability of dissolved inorganic carbon (CO_2_ and HCO_3−_) enhancing inward CO_2_ diffusion rates, consequently increasing carbonate availability in the calcifying fluid (*18*). Our findings also suggest a different fate for high-Mg species, which might act as “first responders” to ongoing OA, as their influence over microenvironmental pH is not as robust as that of their low-Mg/Ca counterparts.

### The influence of H^+^/Ca^2+^ transporters on calcification

The different proton pumping rates observed here may explain differences in elemental composition of these two foraminifers’ calcite. For example, species with low Mg/Ca ratios could rely on Ca^2+^ accumulation within a more closed system and therefore display a higher activity of H^+^/Ca^2+^ transporters, which in turn would lead to a tight control over the pH_cf_. Conversely, species with high Mg/Ca ratios might precipitate their calcite from a fluid more similar to seawater (*43*), making them more susceptible to ambient carbon perturbations.

No direct quantification of calcium fluxes was performed, but according to previous works, *A. tepida* uses a Ca^2+^/H^+^ exchanger (*18*), similar to that in other marine calcifiers (*44*, *45*). In addition, proteomic analysis of calcifying specimens of *A. tepida* indicates the activity of P-type Ca^2+^ transporter type 2B (PMCA) (*19*), while the application of a vacuolar-type (V-type) H^+^ adenosine triphosphatase (ATPase) inhibitor also suggests the activity of this enzyme (*18*). The activity of both these transporters suggests a coupling of H^+^ and Ca^2+^ transport, likely in a 2 (H^+^):1 (Ca^2+^) stoichiometry to maintain electroneutrality (*18*), provided that no other ions are involved. Here, we tested the specificity of this H^+^/Ca^2+^ exchange system (Fig. 2, A to C), demonstrating that the H^+^ efflux depends on the availability of external Ca^2+^. This implies that proton pumping depends on the influx of Ca^2+^. This, in turn, likely sets the saturation state with respect to calcite and the ratio between other elements and Ca^2+^ in the fluid from which the calcite precipitates.

**Fig. 5.**
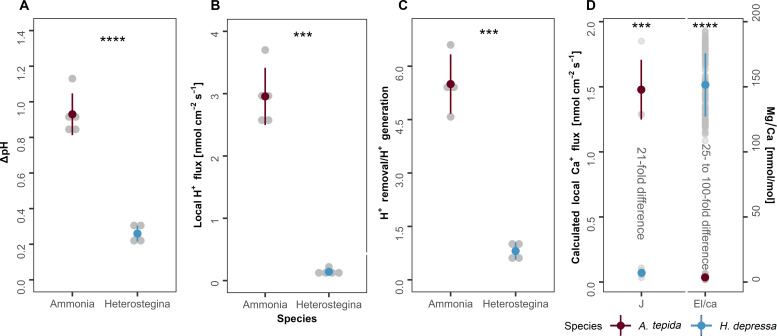
Measured and calculated proton and calcium fluxes. (**A**) The comparison of ΔpH between seawater (100 to 110 μm away from the foraminiferal shell) and microenvironment around the new chamber, (**B**) calculated local H^+^ flux, (**C**) the amount of calcium removed and generated by calcification, and (**D**) the calculated local Ca^+^ flux assuming a 2:1 stoichiometry (*18*) and the calcite’s Mg/Ca ratios of *A. tepida* and *H. depressa* (*23*, *26*, *48*). Significant differences were determined using the unpaired Student’s *t* test. Significance is denoted by *P* ≤ 0.05, ****P ≤ 0.0001, and ***P ≤ 0.001. Red and blue dots and lines represent means + SD values, while gray dots represent bulk values. Complete dataset is available in dataset S1.

The decrease in external pH observed in the studied species (Fig. 1, A and B) can be translated into H^+^ fluxes [see (*18*)], with the calculated local flux being higher in *A. tepida* (*J* = 2.96 ± 0.46 nmol cm^−2^ s^−1^) compared to *H. depressa* (*J* = 0.143 ± 0.06 nmol cm^−2^ s^−1^) (*t* test, *P* < 0.01, *t* = 13, df = 4) (Fig. 5B). For the latter species, total outward H^+^ fluxes varied between 598 and 1470 pmol among specimens (Table 1). The calcified chambers (81 to 125 μm in diameter; Table 1) considering a 3-μm thickness and 25% porosity (*18*) would correspond to a CaCO_3_ production of 58 to 142 ng. Following equimolar transformation of CO_2_ to ${\text{CO}}_{3}^{2-}$ (*18*), H^+^ generation by calcification ranged from 1166 to 2853 pmol. This implies that the amount of H^+^ generated is similar to the amount of H^+^ removed in our analysis (Table 1), indicating a nearly neutral to negative net balance ratio of 0.81 ± 0.24 (H^+^ removal/ H^+^ generation; Fig. 5C) for *H. depressa*. This balance could be efficient in avoiding changes in pH (and Ca^2+^ concentration) due to the ongoing mineralization inside the SOC. However, we note that such measured transmembrane H^+^/Ca^2+^ fluxes may not fully account for all Ca^2+^ uptake during calcification, due to contributions from an internal calcium pool (related to endocytosis) and/or seawater leakage may exist as have been observed in other high-Mg/Ca species like *Amphistegina* spp. (*43*, *46*). Transmembrane transport, if aided by endocytosis and/or seawater transport, might sustain a positive balance ratio of calcium uptake/necessary for calcification, rather than just neutral, as the sole transmembrane ion transport (TMT) would provide (Fig. 5C). Such an increase in calcium concentration is necessary to achieve the optimum conditions at the SOC and induce CaCO_3_ precipitation.

Conversely, the total outward H^+^ fluxes for *A. tepida* varied between 1447 and 9662 pmol (Table 1). Calcified chambers had diameters of 40 to 94 μm producing 13 to 78 ng of CaCO_3_ that correspond to a H^+^ generation of 131 to 785 pmol. Such H^+^ removal exceeds the H^+^ production by calcification by more than five times (with an average ratio of 5.50 ± 0.83) and is significantly higher than in *H. depressa* (*t* test, *P* < 0.01, *t* = 11, *df* = 3.5) and consistent with the species’ need to maintain a steep inward-outward pH gradient. Consequently, the amount of Ca^2+^ taken up for calcification is much higher, assuming a 2:1 stoichiometry: between 724 and 4841 pmol (Fig. 5C). In addition, proteomics have also revealed the expression of voltage-dependent calcium channels and Ca/H antiporter during *A. tepida* calcification, indicating the additional uptake of Ca^2+^ into vesicles (*19*). However, previous CLSM data indicate minor vesicle activity, with 90% of the Ca^2+^ containing vesicles not participating of calcification. This suggests a minor contribution from such a calcium pool in this species, with TMT playing a major role (*47*).

The sole measure of large sustained fluxes of Ca^2+^ and inorganic carbon are sufficient to increase calcification rates as concentrations of all elements, including Mg^2+^, are diluted in SOC relative to that of Ca^2+^. This explains the significant differences in the time taken for each species to calcify (*t* test, *P* < 0.01, *t* = −6.5, df = 3): *A. tepida* (5.74 ± 0.50 hours) and *H. depressa* (18.4 ± 3.89 hours; Table 1). Likewise, since the differences in the total flux of H^+^ and Ca^2+^ ions affect the cation composition of their calcifying fluid, the differences in proton flux (hence Ca^2+^ flux) should also correlate with the substantial offset (25 to 100 times) in the resulting calcite average Mg/Ca ratios. *H. depressa* averages 110 to 140 mmol/mol, while *A. tepida* averages 2 to 7 mmol/mol (*23*, *26*, *48*). We found that the calculated Ca^2+^ ion fluxes exhibited differences of similar magnitude between the two species (~21 times, *t* test, *P* < 0.01, *t* = 14, *df* = 4; Fig. 5D). For *A. tepida*, the rate was *J* = 1.48 ± 0.22 nmol cm^−2^ s^−1^, whereas for *H. depressa*, it was *J* = 0.07 ± 0.03 nmol cm^−2^ s^−1^.

### Mitochondria activity supports transmembrane ion transport

In foraminifera, mitochondria have been observed within rhizopodia and/or cytoplasm adjacent to the organic templates during chamber formation (*21*, *24*, *30*). Their role in calcification, however, may be attributed to the energy demand of proton pumping (*18*), Mg^2+^ and Ca^2+^ removal (*19*), or a combination of both factors.

Here, the close relationship between the timing of mitochondrial movement toward the location where proton pumping is observed and the observed drop in pH outside the foraminifera suggests that the mitochondria provide the energy required by the transmembrane proton transport (Figs. 1 and 3). Although this does not necessarily imply that mitochondrial activity as such increases, a causal link is confirmed by the higher density of mitochondria in the species with a stronger external pH decrease (i.e., *A. tepida*). Such dependence of calcification on mitochondria has also been verified before by inhibition of adenosine triphosphate (ATP) production and transport within mitochondria, confirming that ATP used in calcification is derived from cellular respiration (*49*). In *A. tepida* (Fig. 3C), mitochondrial movement toward the pseudopodia further indicates that H^+^ pumping might also occur within their extensions, which possibly increases the surface area between the cell and the surrounding environment to accommodate the enzymatic “machinery” for calcification. The stronger pH decrease observed in *A. tepida* (Fig. 5, A to F) could potentially be explained by a combination of these two strategic factors: higher mitochondrial activity sustaining the stronger pH modulation and a larger surface area potentially enhancing transmembrane ion transport by creating more space to accommodate additional enzymes.

Concerning *H. depressa* (Fig. 4C), no mitochondrial movement toward the pseudopodia was observed, but mitochondrial networks also accumulated over the edge of the calcifying chamber where modest pH decreases were observed. Such a pattern was not observed during respiration, in which mitochondria were located within the test (fig. S1, A to C).

The ATP requirement of ion transport may signal mitochondria to move toward the SOC, although mitochondrial distribution may also be intrinsically influenced by elevated levels of Mg^2+^ and Ca^2+^ within the protective envelope, which separates the growing calcite surface from the surrounding seawater (*19*). Mg^2+^ plays a critical role in regulating mitochondrial functions, including the stimulation of dehydrogenases and activation of mitochondrial F0/F1-ATPase for ATP synthesis (*50*). Mg^2+^ uptake in mitochondria occurs through high-capacity selective Mrs2 channels (*51*–*53*), which genes are highly expressed in calcifying foraminifera (*19*). Experimental evidence shows that these channels respond positively to an increased external Mg^2+^ concentration (*51*, *53*), and therefore, excess Mg^2+^ at the protective envelope may be absorbed into the mitochondrial matrix, sustaining ATP production and enhancing CaCO_3_ precipitation by down-regulation of Mg^2+^. This is supported by the observation that low-Mg species, such as *A. tepida*, display a higher number of mitochondria associated with calcification compared to their high-Mg counterpart, *H. depressa* (see Figs. 3 and 4). Still, dysregulation of mitochondrial Mg^2+^ can disrupt cellular physiology and energy status (*54*), which may explain why maintaining minimum seawater Mg^2+^ levels (between 1 and 5 mol mol^−1^) is necessary for foraminiferal growth (*55*–*57*).

**Fig. 6.**
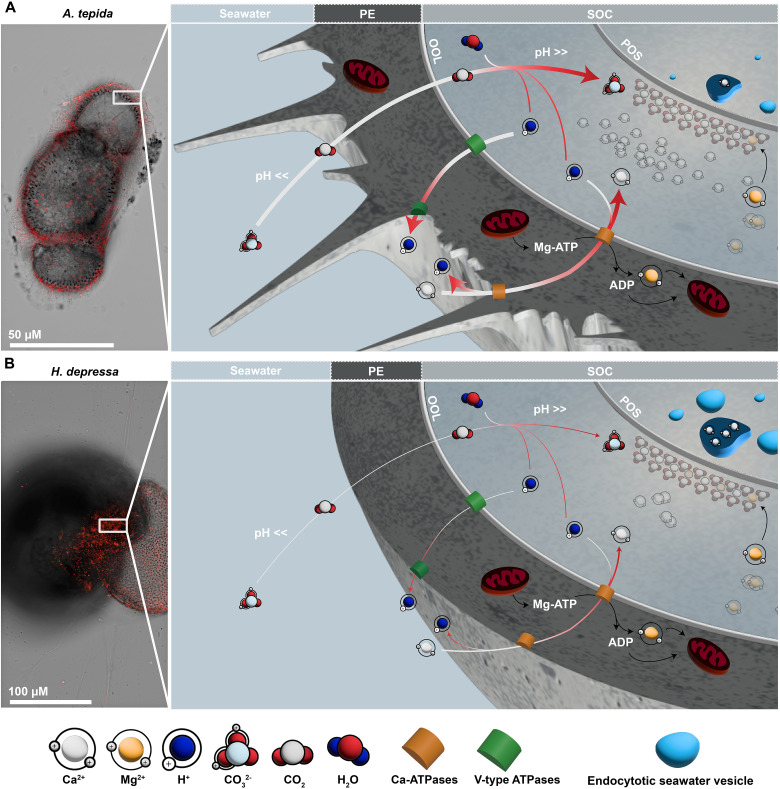
Proposed simplified calcification model for *A. tepida* and *H. depressa*. The external layers involving the SOC are impermeable to (charged) inorganic ions, and the transport of Ca^2+^, inorganic carbon, and H^+^ necessary to induce crystallization is subjected to cellular control via specific transporters and/or membrane channels (*47*). The plasma membrane PMCA pump is responsible for the Ca flux between the seawater and the “privileged space,” in exchange for one proton per ATP molecule hydrolyzed (*65*, *66*). The exact configuration of the proteins responsible for the Ca flux remains to be investigated, but considering the presence of V-type H^+^ ATPase, the PMCA is here considered to exchange one Ca^2+^ for two H^+^ to maintain electroneutrality. The uptake of inorganic carbon, on the other hand, is down-regulated by the activity of Uniport V-type ATPases (*18*). In (**A**) *A. tepida*, the H^+^/Ca^2+^ is higher than in (**B**) *H. depressa*, inducing a stronger pH gradient between the external medium (~6.9 pH units, shown here) and at the SOC [~9 pH units; (*17*)]. This causes a steep pH gradient (low outside, high inside) that facilitates the inorganic carbon uptake by inward CO_2_ diffusion and subsequent conversion to ${\text{CO}}_{3}^{2-}$ ions at the SOC. Concurrently, the higher activity of H^+^/Ca^2+^ transporters increases the uptake/accumulation of Ca^2+^ compared to its removal through calcite crystallization at the SOC. Intracellular Ca^2+^ supply via leakage/vacuolization is likely important in *H. depressa* calcification but is very limited in *A. tepida*, with transmembrane transport playing a predominant role. At the SOC, mitochondrial networks are directly coupled to ATP-dependent enzymatic activities, providing the energy required for the transmembrane transporters. PE, protective envelope; POS, primary organic sheet; OOL, outer organic lining; IOL, inner organic lining; ADP, adenosine diphosphate.

On the basis of these observations, we propose a calcification model (Fig. 6) where Ca^2+^ fluxes and its accumulation within the SOC are coupled to H^+^ electrochemical gradients maintained by V-type H^+^ ATPase activity (*18*). In this model, the final Mg/Ca composition reflects the rate of transmembrane Ca^2+^ transport relative to seawater transport rates (leakage and/or vacuolization). Seawater trapped during the formation of the protective envelope is considered the primary source of ions for calcification, with the boundary between SOC and the surrounding seawater allowing Ca^2+^, C, and H^+^ transport via specific transporters and membrane channels during calcification (*47*). The calcification system is separated from the surrounding seawater in low-Mg species, and it is relatively more open in high-Mg species. In the first case (Fig. 6A), increased enzymatic activity leads to higher Ca^2+^ uptake and accumulation, affecting the calcifying fluids’ Mg/Ca ratio, which is substantially lower than observed at inorganic equilibrium. Seawater leakage/vacuolization may also supply ions for calcification in *A. tepida*, albeit to a lesser extent, with transmembrane ion transport playing a predominant role. On the other hand, for high-Mg species, e.g., *H. depressa* (Fig. 6B), a lower pumping intensity and rate likely result in higher Mg/Ca values in calcite due to reduced Ca^2+^ uptake. Because of its limited control over the isolation from seawater at the SOC, *H. depressa* does not regulate fluid chemistry as effectively as *A. tepida*, particularly affecting the dilution of Mg^2+^ by Ca^2+^ transport. In this case, seawater leakage/vacuolization might play a role as well, with the calculated Ca^2+^ fluxes potentially not accounting for all the calcium used during calcification.

In conclusion, our results support the importance of transmembrane transporters in calcification, providing mechanistic insight into the differential sensitivity of foraminiferal species to acidification and element incorporation (*18*, *23*, *27*, *30*, *41*, *47*, *58*, *59*). We show that differences in shell Mg/Ca ratios of foraminiferal species correlate with the differential activity of H^+^/Ca^2+^ transporters, and how closed the SOC is, suggesting that, similar to corals (*60*, *61*), resilience of species to ongoing OA is closely tied to their ability to calcify within controlled closed systems, emphasizing the importance of pH_cf_ up-regulation in enhancing organismal resistance to OA.

This has important implications for future carbonate budgets, as their net contribution might vary considerably among different groups of calcifiers and even between different foraminiferal groups. Low-Mg species might increase or maintain their production, while high-Mg groups may decrease, with overall carbonate production depending on their proliferation. This, in turn, might affect ocean carbon chemistry and thereby the capacity of the ocean to buffer ongoing anthropogenic CO_2_ release. In addition, we demonstrate a clear correlation between mitochondrial distribution and H^+^ removal during calcification (Figs. 3C and 4C), indicating the functional role of mitochondria in supplying ATP for ion pumping, while it might remove Mg^2+^ within the SOC.

## MATERIALS AND METHODS

### Specimen collection and culture

Observations on biomineralizing foraminifera were performed on two species of benthic foraminifera: *A. tepida*, phylotype T6 (*62*), and *H. depressa*. Individuals of *A. tepida* were collected from surface sediments of an intertidal mudflat (Mokbaai, the Netherlands) and *H. depressa* from rubble sediments of the Indo-Pacific coral reef aquarium in Burgers’ Zoo, Arnhem, the Netherlands (*63*). The samples were transferred to the laboratory and stored in different aquariums containing seawater (salinity: 35, temperature: 18° and 24°C, respectively). Living specimens characterized by pseudopodial activity and colored cytoplasm were transferred to petri dishes for the induction of asexual reproduction. For the latter, specimens were supplied with living *Dunaliella salina* in North Atlantic seawater (salinity: 35). For *A. tepida*, specimens were incubated at 24°C, and for *H. depressa*, specimens were incubated at 26°C in petri dishes, representing an increase of 6 and 2°C, respectively, compared to the temperature of the stock.

### Experimental design

The experiments were carried out with a confocal microscope (LSM980, Zeiss Instruments). Active mitochondria were labeled with the cell-permeant probe MitoTracker Red CMXRos (concentration: 200 nM, Invitrogen) and ambient seawater pH with the pH indicator HPTS (pyranine 8-hydroxypyrene-1,3,6-trisulfonic acid trisodium salt; H1529, Sigma-Aldrich; concentration: 20 μM) (*16*, *18*).

For ambient pH analysis, a 13-step custom calibration curve between fluorescence ratio and seawater pH was first established. Briefly, fluorescent filters were set at λ^405^exc = 395 to 425 nm and λ^488^exc = 460 to 480 nm, and ratiometric pH images were calculated from JPEG images by dividing λ^405^em/λ^488^em + λ^405^em (λem = 510 to 560 nm) for each pixel. To avoid an error, pixel intensities with a zero value (i.e., with no fluorescent signal) were removed from the analysis. A set of solutions with different pH were adjusted by adding NaOH or HCl and manipulated using a syringe to avoid the exchange of CO_2_ between the atmosphere and solutions and hence minimize changes in pH. The solutions’ pH were measured with a pH meter (Thermo Scientific Orion Versa Star) equipped with an electrode (Thermo Scientific ROSS pH electrode). The resulting pH-normalized calibration curve (fig. S2 and dataset S1) is similar to that previously described in the literature (*16*, *18*).

After calibration, a total of nine individuals were incubated in the MitoTracker solution for 1 hour, and 10 individuals were kept in the HPTS-loaded solution during observations under room temperature (23°C). Since mitochondria could be associated with both foraminifera and hosted symbionts, the chlorophyll autofluorescence signal was also investigated (λ^405^exc = 395 to 425 nm, λem = 661 to 672 nm). During observation, individuals were kept at 23°C, salinity 35. Time-lapse images were taken every 3, 5, 10, or 20 min at ×20 magnification. After acquisition, the pH for each pixel in the image was calculated on the basis of the performed calibration. The translated pH maps are evaluated, and the proton fluxes were calculated from a representative pH map following the methodology of Toyofuku *et al.* (*18*). In this process, pH values from the pixels positioned side by side, i.e., within a line from the surface of the calcifying individual to normal seawater values, are further converted to [H^+^]. On the basis of proton concentrations, local radial flux of protons is calculated using Ficks’s law [for detailed information see (*18*)].

In addition, to investigate the specificity of the H^+^/Ca^2+^ exchange system, two more individuals were cultured with HPTS solution containing different [Ca^2+^]. During the calcification process, well after the calcification site had formed and closed, the seawater with normal [Ca^2+^] was replaced by artificial seawater with reduced [Ca^2+^], 10% of the original (*64*). Salinity was kept at 35 by increasing NaCl, while all remaining seawater elements were kept constant. It was hypothesized that high specificity would lead to a slowdown in proton pumping activity due to the low [Ca^2+^] in modified seawater. Conversely, low specificity would allow the pump to function at normal or near-normal levels, as the exchange would not be limited to Ca but could involve other elements such as barium, strontium, or even Mg, which were kept at their normal concentrations.

### Statistical analysis

The unpaired Student’s *t* test (R software) was conducted to determine differences in proton and calcium flux between *A. tepida* and *H. depressa*, with the significant level of 0.05. Data cited in the text refer to the means ± SD.
