# Supplementary material for: Open or closed: pH modulation and calcification by foraminifera
Source: Sci Adv. 2025 May 2;11(18):eadq8425. doi: 10.1126/sciadv.adq8425 (PMC12047421; doi:10.1126/sciadv.adq8425)
Supplement: Supplementary file 1 — Figs. S1 and S2 Table S1 Legend for dataset S1 [file sciadv.adq8425_sm.pdf]

Science Advances
AAAS

Supplementary Materials for

Open or closed: pH modulation and calcification by foraminifera

Daniel François *et al.*

Corresponding author: Daniel François, daniel.do.nascimento.silva@nioz.nl

Sci. Adv. **11**, eadq8425 (2025)
DOI: 10.1126/sciadv.adq8425

The PDF file includes:

Figs. S1 and S2
Table S1
Legend for dataset S1

Other Supplementary Material for this manuscript includes the following:

Dataset S1

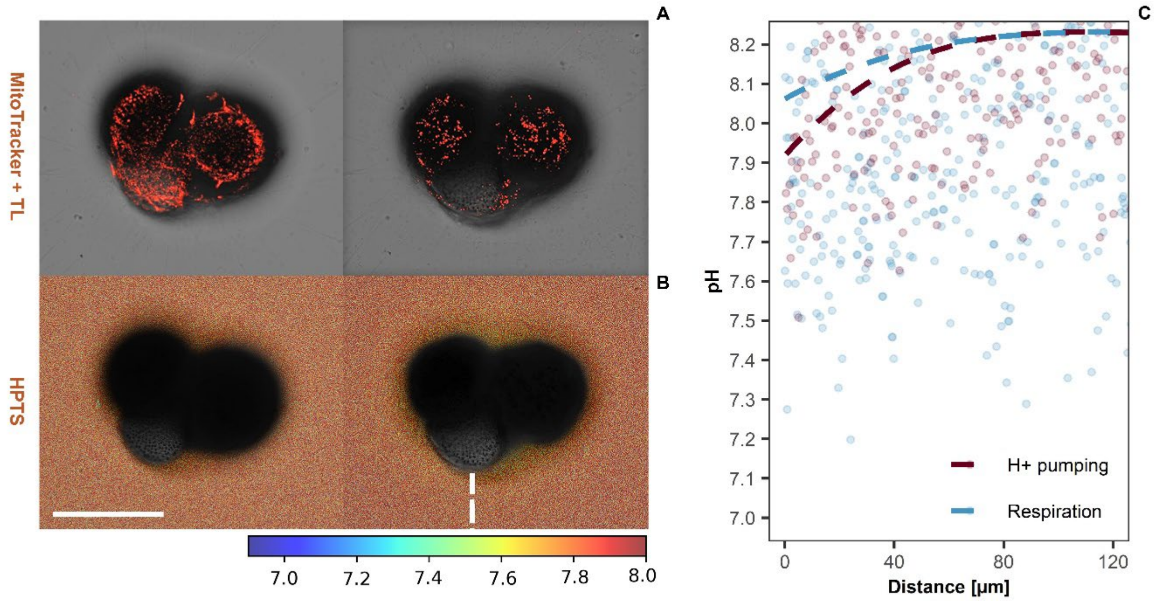

Fig. S1. Mitochondrial and pH maps of *Heterostegina depressa* during respiration. Time series of fluorescence labeling of (A) active mitochondria with MitoTracker Red (200 nM), and (B) seawater pH maps with HPTS (20µM) of *H. depressa* during calcification. The false-color scalebar represents seawater pH changes calculated by dividing λ405em / λ488em + λ405em (λem = 510–560 nm) for each pixel. The artificial red color represents mitochondria labeling. (C) (B) The translated, spatially integrated change in pH measured over the white dashed lines of *Heterostegina depressa* during respiration as shown in (B, blue line) and calcification as shown in Figure 4 (red line).

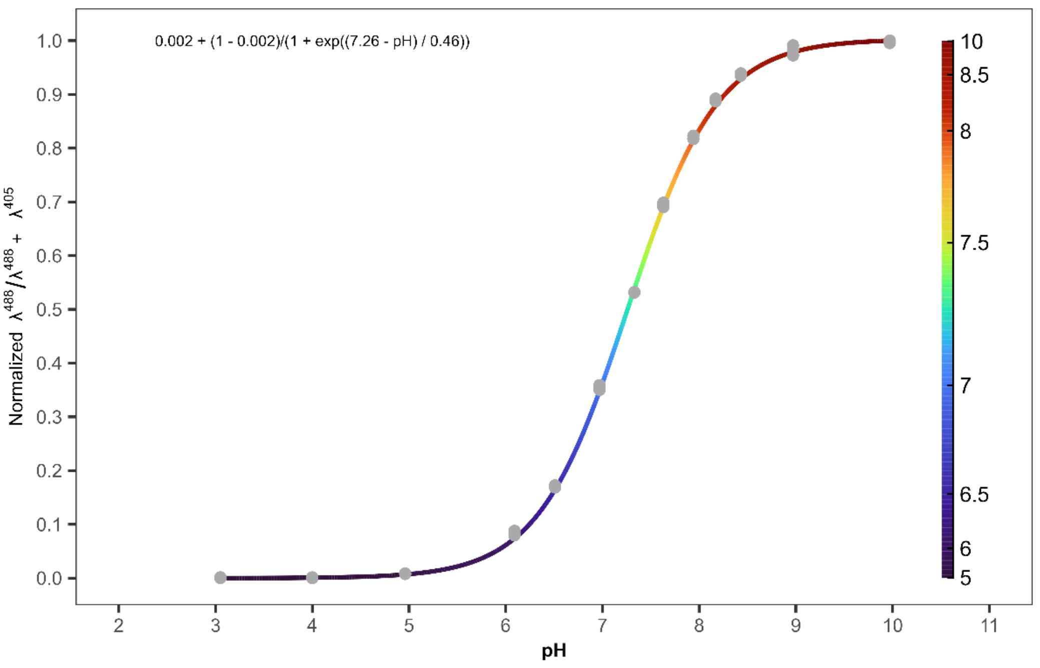

Fig. S2. **13-step custom calibration curve of HPTS (20 µM) as a function of seawater pH conditions**. The relation between λ^{405}em / λ^{488}em + λ^{405}em to seawater pH was found to follow the Boltzmann function.

Individual	Temperature [°C]	Test diameter [µm]	Chamber diameter [µm]	Time	J [nmol/cm^2/s]	Seawater change
1	23	85,39	46,4	17:26	0.07	Before
				17:36	0.38	Before
				17:46	0.61	Before
				17:48	1.01	Before
				17:58	1.54	Before
				18:08	1.49	Before
				18:18	1.81	Before
				18:38	0.15	After
				18:42	0.00	After
				18:52	0.08	After
				19:02	0.02	After
				19:12	0.00	After
				19:22	0.00	After
				19:32	0.00	After
				19:42	0.00	After
				19:52	0.00	After
				20:02	0.09	After
				20:10	0.00	After
				20:20	0.00	After
				20:30	0.09	After
				20:40	0.00	After
				20:50	0.01	After
				21:00	0.00	After
2	23	100	52	16:03	0.21	Before
				16:13	1.08	Before
				16:23	1.25	Before
				16:42	1.53	Before
				16:53	0.52	After
				16:56	0.46	After
				17:06	0.38	After
				17:16	0.35	After
				17:36	0.31	After
				18:06	0.29	After
				18:26	0.19	After
				18:46	0.15	After
				19:06	0.20	After
				19:26	0.00	After
				19:46	0.19	After
				20:06	0.12	After
				20:26	0.00	After
				20:36	0.00	After

Table S1 – The pH imaging observations and calculated outward proton fluxes during chamber formation of *Ammonia tepida* before and after change of seawater [Ca^{2+}].

Caption of Dataset S1 (provided as separate Excel spreadsheet)

Dataset S1: Foraminiferal elemental chemistry, pH-normalized calibration curve and Net proton fluxes. The dataset includes elemental composition data for Ammonia tepida and Heterostegina depressa retrieved from the literature, as well as processed data used to construct the calibration curve and the full dataset on calculated proton fluxes.
